# Estimating the cost-effectiveness of nutrition supplementation for malnourished, HIV-infected adults starting antiretroviral therapy in a resource-constrained setting

**DOI:** 10.1186/1478-7547-12-10

**Published:** 2014-04-27

**Authors:** John R Koethe, Elliot Marseille, Mark J Giganti, Benjamin H Chi, Douglas Heimburger, Jeffrey S Stringer

**Affiliations:** 1 Centre for Infectious Diseases Research in Zambia, Plot 5032 Great North Road, Lusaka, Zambia; 2 Division of Infectious Diseases, Vanderbilt University School of Medicine, 215 Light Hall, Nashville, TN 37232, USA; 3 Department of Biostatistics, Vanderbilt University School of Medicine, 215 Light Hall, Nashville, TN 37232, USA; 4 Health Strategies International, 555 59th Street, Oakland, CA 94609, USA; 5 Department of Obstetrics and Gynecology, UNC Global Women’s Health, University of North Carolina at Chapel Hill School of Medicine, 3009 Old Clinic Building CB #7570, Chapel Hill, NC 27599-7570, USA; 6 Vanderbilt Institute for Global Health, 2525 West End Ave., Nashville, TN 37203, USA

**Keywords:** HIV, Antiretroviral therapy, Nutrition, Malnutrition, Zambia, Africa

## Abstract

**Background:**

Low body mass index (BMI) individuals starting antiretroviral therapy (ART) for HIV infection in sub-Saharan Africa have high rates of death and loss to follow-up in the first 6 months of treatment. Nutritional supplementation may improve health outcomes in this population, but the anticipated benefit of any intervention should be commensurate with the cost given resource limitations and the need to expand access to ART in the region.

**Methods:**

We used Markov models incorporating historical data and program-wide estimates of treatment costs and health benefits from the Zambian national ART program to estimate the improvements in 6-month survival and program retention among malnourished adults necessary for a combined nutrition support and ART treatment program to maintain cost-effectiveness parity with ART treatment alone. Patients were stratified according to World Health Organization criteria for severe (BMI <16.0 kg/m^2^), moderate (16.00-16.99 kg/m^2^), and mild (17.00-18.49 kg/m^2^) malnutrition categories.

**Results:**

19,247 patients contributed data between May 2004 and October 2010. Quarterly survival and retention were lowest in the BMI <16.0 kg/m^2^ category compared to higher BMI levels, and there was less variation in both measures across BMI strata after 180 days. ART treatment was estimated to cost $556 per year and averted 7.3 disability-adjusted life years. To maintain cost-effectiveness parity with ART alone, a supplement needed to cost $10.99 per quarter and confer a 20% reduction in both 6-month mortality and loss to follow-up among BMI <16.0 kg/m^2^ patients. Among BMI 17.00-18.49 kg/m^2^ patients, supplement costs accompanying a 20% reduction in mortality and loss to follow-up could not exceed $5.18 per quarter. In sensitivity analyses, the maximum permitted supplement cost increased if the ART program cost rose, and fell if patients classified as lost to follow-up at 6 months subsequently returned to care.

**Conclusions:**

Low BMI adults starting ART in sub-Saharan Africa are at high risk of early mortality and loss to follow-up. The expense of providing nutrition supplementation would require only modest improvements in survival and program retention to be cost-effective for the most severely malnourished individuals starting ART, but interventions are unlikely to be cost-effective among those in higher BMI strata.

## Background

The rapid expansion of access to combination antiretroviral therapy (ART) for the treatment of HIV infection in sub-Saharan Africa over the past decade has occurred against a backdrop of persistent poverty, underdevelopment, and widespread food insecurity [1,2]. Inadequate protein and energy intake accelerates HIV-associated weight loss and immunosuppression, and a low body mass index (BMI; <18.5 kg/m^2^) at treatment initiation is associated with 2 to 6 fold increased mortality within 90 days compared to higher BMI in several analyses from the region [3-6]. In an analysis from Zambia, 20% of persons with a pre-treatment BMI <16 kg/m^2^, indicative of severe malnutrition by World Health Organization (WHO) criteria, were deceased after 6 months of ART and an additional 18% were lost to follow-up [7]. While the etiology of these early deaths is poorly understood, weight gain in the immediate post-ART period is a predictor of long-term survival in low BMI adults, suggesting that interventions to promote nutritional rehabilitation on ART may impact long-term outcomes [7-9].

The few published trials of macronutrient supplementation (i.e., products containing protein, carbohydrate or fat sources rather than vitamins or minerals alone) found heterogeneous effects on early ART adherence, client retention, and health outcomes [10-15]. However, the provision of supplementation at a program level will require additional expenditures and resource allocation at a time when many countries are still expanding to meet the need for ART services. In lieu of a large prospective trial, cost-effectiveness modeling provides a framework to assess the opportunity costs and potential benefits of a range of prevention, treatment and care options, and can be used to estimate the feasibility of introducing a new intervention using available data. In this analysis, we use cost estimates from a recent analysis of 45 ART clinics in Zambia, in combination with health outcomes data on over 19,000 patients, to model the minimum improvements in survival and program retention necessary for a hypothetical combined nutrition support and ART treatment program to maintain cost-effectiveness parity with ART treatment alone [16].

## Methods

The setting for this analysis was the Zambian national program for HIV care and treatment, implemented in April 2004 [3,17]. We constructed competing Markov models to assess the circumstances under which the cost per disability adjusted life year (DALY) averted resulting from the addition of a nutrition supplement for 6 months as an adjunct to ART treatment would be equally or more cost-effective than standard treatment with ART. We selected a 6 month intervention based on epidemiologic studies from Zambia and other countries in sub-Saharan Africa demonstrating that mortality rates are markedly higher among low-BMI adults in the first 6 months of ART treatment, and then normalize compared to higher BMI groups after this period [4,7,8].

Our primary estimates of ART program costs and health outcomes are drawn from a program-wide cost-effectiveness analysis of HIV treatment and care in Zambia, which tabulated costs at 45 public and private-sector health centers from April 2004 through July 2008, and reported facility-level unit costs as the full incremental cost of providing ART divided by the number of person-years on treatment [16]. We used this analysis as our base estimate for two major reasons; first, many of the clinical sites surveyed served the same patients comprising the historical cohort used to derive our mortality and retention data. Second, the HIV epidemic in Zambia was characterized by a high prevalence of advanced disease (i.e., AIDS) and an under-developed health system in the early stages of the rapid scale-up of ART services beginning in 2004, which may have had unique effects on both program costs and health effects. However, we also performed a sensitivity analysis around the base ART cost estimate using recent systematic reviews from the region [18-21].

Our data on mortality and loss to follow-up over the first 6 months of ART among malnourished adults starting ART in Zambia are derived from a historical cohort of 58,380 adults (19,247 with BMI <18.5 kg/m^2^) receiving ART in Zambia from May 2004 through October 2010. These data were generated by the custodian of the national ART program database for the purpose of this analysis and stratified by WHO criteria: BMI <16.0 kg/m^2^ (severe malnutrition), 16.00-16.99 kg/m^2^ (moderate malnutrition), and 17.00-18.49 kg/m^2^ (mild malnutrition) [22].

**Figure 1:**
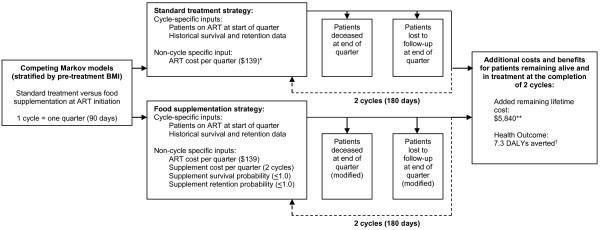
**Design of the cost-effectiveness model.** *ART cost per quarter calculated as the average cost per ART year ($556) divided by four. This figure represents year one costs and is not subject to discounting. **Added lifetime cost per active ART patient is calculated by subtracting cumulative ART cost accrued during 2 model cycles from an overall cost of $6,118 per death averted, or the lifetime expenditure per patient (discounted at a rate of 3% and accounting for historical rates of second-line ART use). Cost estimates for ART and the switch rate from first to second-line treatment are from a PEPFAR-supported, program-wide cost-effectiveness analysis of 45 public and private-sector centers providing HIV treatment in Zambia between 2004 and 2008.

We used Markov models to portray disease progression through a series of distinct states; in our model, individuals were (1) alive and active in care, (2) deceased, or (3) lost to follow-up from the treatment program. Transition state probabilities were drawn from the historical cohort data and were unidirectional; patients in the deceased and lost to follow-up terminal states could not transition back to the alive and active in care state (a separate sensitivity analysis modeled patients classified as lost at 6 months who subsequently returned to the program). Each model cycle represented a 90 day period, and patients remaining alive and active in care at the completion of 2 model cycles (approximately 6 months) were assigned a fixed lifetime program cost and health outcome. We modeled the potential effect of nutrition supplementation on the rate of transition to the deceased or lost to follow-up states using survival and retention probabilities, which represent projected proportional reductions in historical mortality and loss to follow-up rates as a result of implementing a supplementation program. Figure 1 describes the model design.

The ratio of program cost to DALYs averted with ART treatment in each BMI stratum was calculated using the historical survival and retention rates, which provided the *willingness to pay* (WTP) value. The WTP value represents the benchmark cost-effectiveness for assessing combinations of supplement costs and relative reductions in survival and/or loss to follow-up. If the program cost per DALY averted for a given combination of supplement cost and survival/retention improvement exceeds the WTP value of ART alone, the combination is considered less cost effective than standard ART provision.

Using the WTP values for each BMI stratum as a threshold, we calculated the maximum quarterly supplement costs permitted to maintain parity with ART treatment alone for four hypothetical scenarios: first, if the added costs of supplement conferred only a 20% reduction in mortality; second, only a 20% reduction in loss to follow-up; third, a 20% reduction in both endpoints; or fourth, a 50% reduction in both endpoints. We then compared the maximum permitted quarterly supplement costs for each of these four scenarios against the cost of providing half of an HIV patient’s recommended daily caloric intake for 90 days using several supplements common to the region.

Lastly, to demonstrate the effects of selecting a more expensive supplement product over a less expensive product, we graphed the effects of interval increases in quarterly supplement costs against the necessary change in proportional reduction in mortality and/or loss to follow-up required to maintain parity with ART treatment alone in each low BMI stratum.

### Clinical estimates

#### ***Survival***

Baseline quarterly estimates of survival on ART were drawn from a historical cohort of 58,380 adults (19,247 with BMI <18.5 kg/m^2^) initiating treatment at Lusaka district health clinics between May 1, 2004 and October 1, 2009, with clinical follow-up through October 1, 2010 (Table 1). BMI-stratified hazard ratios for death were calculated at 90 day intervals from ART initiation to 360 days.

**Table 1:** Clinical and cost estimates in the cost-effectiveness model

Historical quarterly survival and loss to follow-up in the Zambian national ART program from May 2004 through October 2010 stratified by BMI*					
**BMI (kg/m**^**2**^**)**	**Quarter**	**Patients alive/active at start of quarter**	**Disposition at end of quarter (%/cumulative %)**		
			**Alive/active**	**Dead**	**Loss to follow-up**
<16.0	Q1; 0-90 days	5096	3649 (71.6 / na)	881 (17.3 / na)	566 (11.1 / na)
	Q2; 91-180 days	3649	3131 (85.8 / 61.4)	181 (5.0 / 20.8)	337 (9.2 / 17.7)
	Q3; 181-270 days	3131	2875 (91.8 / 56.4)	73 (2.3 / 22.3)	183 (5.8 / 21.3)
	Q4; 271-360 days	2875	2689 (93.5 / 52.8)	40 (1.4 / 23.1)	146 (5.1 / 24.2)
16.00 - 16.99	Q1	4439	3608 (81.3 / na)	468 (10.5 / na)	363 (8.2 / na)
	Q2	3608	3222 (89.3 / 72.6)	124 (3.4 / 13.3)	262 (7.3 / 14.1)
	Q3	3222	2995 (93.0 / 67.5)	45 (1.4 / 14.4)	182 (5.7 / 18.2)
	Q4	2995	2813 (93.9 / 63.4)	34 (1.1 / 15.1)	148 (4.9 / 21.5)
17.00 - 18.49	Q1	9712	8421 (86.7 / na)	631 (6.5 / na)	660 (6.8 / na)
	Q2	8421	7630 (90.6 / 78.6)	228 (2.7 / 8.8)	563 (6.7 / 12.6)
	Q3	7630	7177 (94.1 / 73.9)	103 (1.4 / 9.9)	350 (4.6 / 16.2)
	Q4	7177	6849 (95.4 / 70.5)	58 (0.8 / 10.5)	270 (3.8 / 19.0)
>18.5	Q1	39133	35716 (91.3 / na)	1229 (3.1 / na)	2188 (5.6 / na)
	Q2	35716	33417 (93.6 / 85.4)	460 (1.3 / 4.3)	1839 (5.1 / 10.3)
	Q3	33417	31850 (95.3 / 81.4)	251 (0.8 / 5.0)	1316 (3.9 / 13.7)
	Q4	31850	30432 (95.5 / 77.8)	200 (0.6 / 5.5)	1218 (3.8 / 16.8)
**Cost estimates (all BMI strata)**					
ART treatment			$556 per year ($139 per quarter)		
**Health outcomes estimate (all BMI strata)**					
Health benefits per AIDS death averted			7.3 Disability-adjusted life years		

*Derived from a historical cohort of 58,380 patients initiating ART in Lusaka district health clinics between May 1, 2004 and October 1, 2009; 19,247 (33%) had a baseline BMI <18.5 kg/m^2^.

*Abbreviations*: *ART* antiretroviral therapy, *BMI* body mass index.

#### ***Retention***

Baseline quarterly estimates of program retention were calculated using the same historical cohort. Loss to follow-up was defined according to the internal programmatic definition of no return to clinic within 37 days after a scheduled pharmacy visit or within 60 days of the last clinical visit if no pharmacy visit was scheduled. A sensitivity analysis assessed the effect of varying proportions of patients classified as lost to follow-up subsequently returning to the ART program.

#### ***Health outcomes***

Cost-effectiveness was portrayed as the program cost per DALY averted, an indicator of the time lived with a disability and the time lost due to premature mortality [23]. Treatment with ART was estimated to avert 7.3 discounted DALYs among HIV-infected individuals who survived >16 weeks. This value was drawn from the same Zambian analysis used to estimate treatment costs, and it was obtained by comparing deaths in the Zambian ART program with deaths observed in a prior, well-characterized home-based AIDS care (HBAC) cohort of 466 HIV-infected patients not receiving ART in Tororo District, Uganda, adjusted for differences in CD4+ T-cell counts and sex distribution between groups [16,19,24,25].

### Cost estimates

#### ***First-line ART***

The unit cost estimate for antiretroviral agents and ART-related health care per person-year (i.e., an ART-year) was drawn from the cost-effectiveness analysis of 45 treatments sites across Zambia [16]. The sites were predominantly publically-funded (87%), urban (84%), and located in clinics (64%) as opposed to hospitals. The overall program cost was estimated to be $69.7 million for 125,436 person-years of ART delivered between April 26, 2004 and July 1, 2008, or a pooled average of $556 per ART-year (adjusted to 2010 US dollars according to the consumer price index).The cost per death averted, or the lifetime program cost per patient was $6,118 (discounted at a rate of 3% and accounting for historical rates of second-line ART use).

While our primary cost estimate is similar to another recently published analysis from Zambia which found a cost of $518 to $523 per year for the use of efavirenz and non-generic tenofovir and emtricitabine (the recommended first-line NRTI combination in Zambia since 2007), there was also a high degree of heterogeneity in the average total (i.e., on site and off site) cost at the 45 sites used to derive our estimates, which explained 78% and 66% of the variation of average total and on-site costs respectively [16,18]. For this reason, we also conducted a sensitivity analysis incorporating cost estimates from recent systematic reviews of similar low income countries [20,21].

Our analysis did not consider direct non-health care costs and hospitalization costs, which are an important and often expensive component of overall health care expenditures. First, while we have rigorously collected data on ART costs and survival/retention from the Zambia ART program, we do not have similarly rigorous data on per-patient health care costs for end-stage AIDS, hospice costs, and other end-of-life costs in Zambia. Given the multitude of non-governmental organizations and other groups supporting the health infrastructure in Zambia during the study period, it would be difficult to estimate the proportion of costs that were actually borne by the public sector. Second, we are primarily concerned with the implementation of a nutrition supplementation program within the funding framework of a dedicated ART treatment program. Such programs, often supported heavily by international donors such as the Presidents Emergency Plan for AIDS Relief (PEPFAR), are primarily designed for the delivery of ART services and may not support other health infrastructure including general facilities and hospice care.

#### ***Nutrition supplements***

The range of nutrition supplement costs included in our analysis was informed by contemporary prices for staple commodities consumed in the region [26] and common macronutrient supplements utilized by the World Food Program (WFP) such as corn-soya blend flour (CSB) [26] and Ready-to-Use Therapeutic Food (RUTF) [27,28] (Table 2). Cost estimates were drawn from the literature and United Nations Food and Agricultural Organization historical global food prices [12,29]. The quarterly cost of each supplement was based on the quantity necessary to supply a daily target of 1,360 kcal, which represents 50% of the WFP recommended minimum daily intake of 2100 kcal for adults, increased by an additional 30% (the upper limit of the estimated increase in resting metabolic rate in advanced HIV infection) [30,31]. Because nutrition supplementation is only modeled for the first 6 months of ART, costs were not discounted.

**Table 2:** Comparison of nutritional content and estimated costs of potential supplements

	**Ready-to-use therapeutic food **[27]**,**[28]	**Corn-soya blend flour **[26]	**Common sub-Saharan African staple foods **[26]	
			**Maize meal (yellow)**	**Rice (white)**
Grams:	100	100	100	100
Calories:	557	376	366	365
Protein grams (% kcal):	14 (10%)	17 (18%)	8.5 (9%)	7.1 (8%)
Fat grams (% kcal):	35 (59%)	7.0 (17%)	1.7 (4%)	0.7 (2%)
Carbohydrate % kcal:	31%	65%	87%	90%
Indication:	Moderate to severe malnutrition	Mild to moderate malnutrition	Staple food	Staple food
Ingredients:	Plumpy’Nut: vegetable fat, peanut paste, skimmed milk powder, whey powder, malto-dextrin, sugar, mineral and vitamin complex.	Corn and soy blend flour, soybean oil, mineral and vitamin complex.		
Kilograms needed to supply 1,360 kcal/day for 3 months*	21.97	32.55	33.44	33.53
Estimated cost per kilogram (USD)	$2.18†	$0.48†	$0.296‡	$0.540‡
Estimated cost to provide 1,360 kcal/day for 3 months (USD)^Ω^	$47.89	$15.66	$9.90	$18.11

Note: cost-effectiveness models provide a threshold value for supplement costs, however the selection of a specific product would be highly dependent on the local cost of delivering the supplement to patients.

*The figure of 1,360 kcal/day represents 50% of the WFP recommended minimum daily intake of 2100 kcal for adults, increased by an additional 30% (the upper limit of the estimated increase in resting metabolic rate in advanced HIV infection).

†Reported costs per kilogram for locally produced, peanut-based ready-to-use spread and corn-soya blend flour from a nutrition supplementation trial in Malawi by Ndekha *et al*. [12].

‡Annual average price in 2012 for US #2 yellow maize and white broken rice (Thai A1 Super). Source: Food and Agriculture Organisation of the United Nations (http://www.fao.org/es/esc/prices). Current prices may be higher.

^Ω^Excludes all external costs (transport, storage, etc.).

### Sensitivity analyses

We performed sensitivity analyses around ART program costs and the effect of patients classified as lost to follow-up subsequently returning to care. Both sensitivity models assume a 20% mortality and 20% loss to follow-up reduction from the supplement to calculate the maximum supplement costs which would be cost-effective compared to standard treatment across a range of input values. To assess the effect of ART program costs, we utilized estimates from a recent systematic review of six studies in four low income countries that found a median cost per ART patient-year of $792 and a range of $682 to $1,089, which was similar to other systematic reviews from the region [20,21,32]. The upper bound of this range is approximately 196% of our estimate of $556 per year, and the sensitivity analysis considers ART costs ranging from $500 to $1200 per patient-year ($125 to $300 per quarter).

Our primary analysis treats patients lost to follow-up in the first 6 months of treatment as permanent exits from the HIV program, and while a large systemic review found approximately 40% of African ART patients lost to follow-up early in treatment represent unrecorded deaths, a proportion of the lost patients will eventually return to care [33]. For this reason, we modeled the effect of 20% to 80% of patients classified as lost subsequently returning to care and contributing to lifetime ART costs and DALYs averted (we assumed they would not receive a second course of supplementation).

### Analysis software

Survival and retention estimates were calculated from the historical cohort data using SAS version 9.1 (SAS Institute, Cary, North Carolina). Markov modeling and cost-effectiveness analyses were performed using TreeAge Pro Healthcare module (Version 2014, TreeAge Software, Inc. Massachusetts, USA).

## Results

### Survival and retention in the historical cohort

Among 58,380 patients starting ART in Lusaka district health clinics between May 1, 2004 and October 1, 2009, 19,247 (33%) had a BMI <18.5 kg/m^2^ at treatment initiation. Among those with BMI <16.0 kg/m^2^, 17.3% were deceased before 90 days and 20.8% by 180 days, while 11.1% and 17.7% were classified as lost to follow-up at the same time points (Table 1). In contrast, 3.1% of patients with a BMI >18.5 kg/m^2^ were deceased at 90 days, and 5.6% were lost to follow-up. Quarterly survival and retention generally improved as BMI increased, and there was minimal variation in rates between BMI categories after 180 days of treatment. Within the malnourished BMI strata, the BMI <16.0 kg/m^2^ group had a slightly higher proportion of women compared to the BMI 17.00-18.49 kg/m^2^ group (59% versus 53%), lower median age (33 versus 35 years), and a lower median CD4+ T-cell count (92 versus 127 cells/μl).

### Effect of changes in survival and retention probability on cohort health outcomes

**Figure 2:**
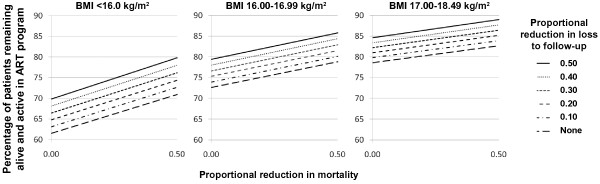
**Estimated percentage of patients remaining alive and on antiretroviral therapy at 6-months as a result of proportional reductions in the historical quarterly death and loss to follow-up rates, stratified by BMI.** The effect of a proportional reduction in quarterly loss to follow-up rates is represented by the diagonal lines, and the effect of a reduction in quarterly mortality rates is shown on the x-axis. Locate a mortality reduction value (0 to 50%) on the given loss to follow-up reduction line, and the percentage of patients estimated to remain alive and on ART at 6 months as a result of the assumed changes is shown on the y-axis. The historical program retention rate corresponds to the y-intercept of the lowest diagonal line (i.e., no assumed reduction in loss to follow-up). Abbreviations: ART, antiretroviral therapy; BMI, body mass index.

The anticipated effect on overall 6 month treatment outcomes resulting from a proportional reduction in the historical quarterly mortality and loss to follow-up rates (modeled as zero to 50%) varied across the BMI strata (Figure 2). The projected improvements in the number of patients alive and active in care was most pronounced in the BMI <16.0 kg/m^2^ category; for every 100 patients initiating ART, approximately 18 additional individuals were estimated to remain alive and active in care at 6 months when both the historical mortality and loss to follow-up rates were reduced by 50%. In the BMI 17.00-18.49 kg/m^2^ category, similar reductions resulted in approximately 10 more patients alive and in care at 6 months.

### Cost-effectiveness requirements for nutritional supplement intervention

The WTP values, or the benchmark cost-effectiveness of standard ART treatment against which alternate interventions can be compared, were $853.31, $847.53, and $845.87 per DALY averted in the BMI <16.0, 16.00-16.99, and 17.00-18.49 kg/m^2^ categories, respectively. The WTP values varied across the BMI categories as a function of the historical mortality and loss to follow-up rates in each category; because a greater proportion of BMI <16.0 kg/m^2^ persons accrued early ART costs but ultimately failed to survive, the cost per DALY averted is higher in this group.

Using the WTP values for each BMI stratum, we modeled four hypothetical scenarios: first, if the added costs of supplement conferred only a 20% reduction in mortality; second, if the supplement conferred only a 20% reduction in loss to follow-up; third if the supplement conferred a 20% reduction in both endpoints; or fourth, if the supplement conferred a 50% reduction in both endpoints (Table 3). Based on published costs in the literature and United Nations Food and Agricultural Organization historical global food prices, the provision of 1360 kcal/day for 3 months was estimated to cost $9.90 using maize flour, $15.66 for CSB, and $47.89 for RUTF in 2012 (Table 2) [12,29]. Therefore, the provision of maize supplement to individuals with a BMI <16.0 kg/m^2^ would be cost-effective if accompanied by a combined 20% reduction in mortality and 20% reduction in loss to follow-up, while the provision of CSB would be cost-effective if accompanied by a 50% combined reduction. In the BMI 16.00-16.99 kg/m^2^ category, supplementation with CSB was cost-effective if accompanied by a combined 50% reduction in mortality and loss to follow-up, while in the 17.00-18.49 kg/m^2^ category only maize would be cost-effective if the same endpoint was achieved. While our models provide a threshold cost-effectiveness value for the intervention, the selection of a specific product would be highly dependent on the local cost of delivering the supplement to patients. In particular, local mass production of RUTF might be considerably less expensive compared to costs reported in clinical studies [12].

**Table 3:** Maximum allowable quarterly supplement costs to maintain parity with ART alone

**BMI category**			
**Change in overall survival and retention at 6 months of ART with supplement**	**<16.0 kg/m**^**2**^	**16.00-16.99 kg/m**^**2**^	**17.00-18.49 kg/m**^**2**^
**20% mortality benefit, no retention benefit**	$5.49	$3.03	$1.94
**No mortality benefit, 20% retention benefit**	$5.48	$3.83	$3.23
**20% mortality and 20% retention benefit**	$10.99*	$6.86	$5.28
**50% mortality and 50% retention benefit**	$27.48†	$17.20†	$13.01*

*scenarios in which maize flour supplementation would be cost-effective.

†scenarios in which maize flour or corn-soya blend flour supplementation would be cost-effective.

**Figure 3:**
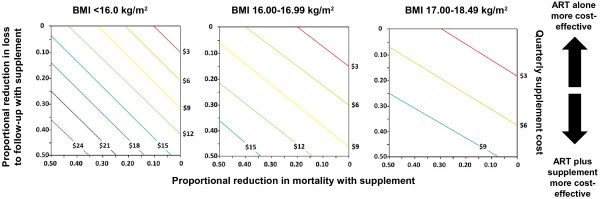
**Proportional reductions in mortality and loss to follow-up necessary to maintain parity with antiretroviral therapy alone for different quarterly supplement costs, stratified by BMI.** Quarterly supplement costs are represented by diagonal lines. Projected reductions in the historical quarterly mortality and loss to follow-up rates are plotted on the x-axis and y-axis, respectively. For a given quarterly supplement cost, the combination of the proportional reduction in mortality and loss to follow-up would need to fall below the diagonal line to be more cost-effective than ART treatment alone. Abbreviations: ART, antiretroviral therapy; BMI, body mass index.

Lastly, we plotted interval increases in quarterly supplement costs against the proportional reduction in mortality and/or loss to follow-up necessary to maintain parity with ART treatment alone in each low BMI stratum (Figure 3). For each quarterly supplement cost value, the accompanying reduction in mortality and loss to follow-up would need to fall below the diagonal line to be more cost-effective than standard treatment. The range of quarterly supplement prices that would be considered cost-effective narrowed markedly as BMI increased. Interventions resulting in a 20% reduction in both mortality and loss to follow-up were cost-effective in the BMI <16.0 kg/m^2^ stratum if less than approximately $10 per quarter or less than $5 per quarter in the BMI 17.00-18.45 kg/m^2^ stratum, while the maximum permitted intervention costs in the model, corresponding to a combined 50% survival and retention improvement, were approximately $26 and $12 per quarter, respectively.

### Sensitivity analyses

#### ***ART program costs***

We modeled the effects of ART program patient-year costs ranging from $500 to $1200 (90% to 216% of our base estimate) on the cost-effectiveness of a supplement conferring a 20% reduction in both survival and retention (Additional file 1: Table S1 and Additional file 2: Figure S1). In the BMI <16 kg/m^2^ stratum, the maximum permitted quarterly supplement cost rose from $10.99 to $23.83 if the cost per patient-year of ART was increased from our base estimate of $556/year to $1200/year. A similar effect was observed in the higher BMI strata, but the magnitude of the increase in permitted supplement costs was not as pronounced as ART program costs increased.

#### ***Return to care of patients classified as lost to follow-up***

We modeled the effect of 20% to 80% of patients classified as lost to follow-up at 6 months subsequently returning to the ART program and resuming care (Additional file 3: Table S2 and Additional file 4: Figure S2). We utilized our base ART cost ($139/quarter) and assumed returned patients would generate the same lifetime ART costs and DALYs averted as patients remaining in care at 6 months from initial ART start (returned patients would not receive a second course of supplementation). The model assumes a 20% reduction in both survival and retention from the intervention. As expected, the maximum permitted supplement cost decreased as more patients returned to care, with the greatest decline in the BMI <16 kg/m^2^ stratum (the group with the highest loss to follow-up rate).

## Discussion

Our analysis of the effects of a nutrition supplementation program for HIV-infected, low BMI adults starting ART in a resource-constrained setting incorporated historical health outcomes data with program-wide cost data to define minimum improvements in survival and program retention necessary to maintain cost-effectiveness parity with ART treatment alone. A 6-month supplementation program was potentially cost effective in comparison to standard treatment in all categories of clinical malnutrition, but the permitted supplement costs were markedly lower for patients with higher pre-treatment BMI. Our results can be used to set retention and survival targets, and an acceptable supplement cost range, for future prospective macronutrient intervention trials to reduce the startlingly high early mortality rate in this population.

In designing a trial of nutrition supplementation, costs should be balanced against reasonable assumptions about the anticipated effects. The incremental reduction in mortality and/or loss to follow-up necessary to justify an interval rise in quarterly supplement costs was smallest in the BMI <16.0 kg/m^2^ category, suggesting that more severely malnourished individuals may be a preferred population for future trials if cost-effectiveness is a concern. However, while cost is an important input variable, the effectiveness of a supplement for achieving the desired endpoint is equally important. Additional studies to characterize the nutritional deficits present in HIV-infected adults with low BMI are needed to determine the optimal supplement composition. For example, the provision of carbohydrate-rich, low-cost local staple foods, such as cornmeal, rice, or green banana, may appear to be the preferred intervention to reduce the supplement cost and maximize cost-effectiveness. However, a predisposition to protein catabolism (especially skeletal muscle) over adipose tissue in HIV infection [34], and an elevated rate of protein turnover [35,36], suggest that low-protein foods may perform poorly for nutritional rehabilitation of severely wasted patients with significant muscle mass reduction [37]. While RUTF is considerably more expensive than maize, RUTF contains far higher fat and protein, which may be of benefit to persons with advanced muscle wasting. Similarly, CSB provides a higher percentage of calories from protein and fat compared to local staple foods at a much lower cost than RUTF, and may represent a cost-effective option for patients in the BMI <16.0 kg/m^2^ stratum if the survival and retention benefits are in the 10-15% range.

A potential limitation of our analysis was the reliance on a single DALY estimate drawn from prior studies of ART cost-effectiveness in Zambia and Uganda, which utilized disability weights from the 2002 Global Burden of Disease (GBD) study [38]. The more recent disability weights from the 2010 GBD study approximate the values used to derive our estimate of 7.3 DALYs averted by ART treatment. We used a disability weight of 0.06 for calculating the benefits of ART versus 0.053 in the 2010 GBD report, an estimate of 0.505 for AIDS not receiving ART versus 0.547, and an estimate of 0.221 for untreated HIV prior to the development of AIDS versus 0.135 [39]. The major difference between the 2002 GBD disability weights used in our analysis and the 2010 update relate to untreated HIV prior to the development of AIDS. However, in the cohort used to derive our estimates, which was typical of many sub-Saharan African countries at the time, advanced HIV disease (i.e. AIDS) was far more common among those presenting for treatment than early-stage HIV. In a sensitivity analysis applying the 2010 GBD disability weights to the Zambian ART program analysis, we found the estimated DALYs averted by ART treatment changed by only 2.6% [16].

Our analysis did not consider several factors which could bias the results by either underestimating or overestimating the cost-effectiveness of nutrition supplementation. While we included the direct costs of HIV care and ART medications at facilities supported by PEPFAR, we did not consider the additional public-sector health costs associated with patients lost to follow-up and admitted to a hospital with advanced disease. Additionally, our approach may have underestimated the additional costs incurred by low BMI patients who ultimately survive as a result of nutrition supplementation but require costly additional monitoring or care in the early post-ART period. Our model also did not address the effect of nutrition supplementation on ART adherence, which has been reported in prior studies and could lead to improved maintenance of viral suppression and reduced costs for expensive second-line agents [10,15].

## Conclusions

In a population of HIV-infected malnourished adults in Zambia, the minimum proportional reductions in mortality and loss to follow-up necessary for a combined nutrition support and ART treatment program to maintain cost-effectiveness parity with ART alone appear to be reasonable endpoints for prospective studies utilizing several candidate supplements. Our analysis methodology can be used to evaluate the cost-effectiveness of future trials of macronutrient supplementation to reduce the high early mortality rate among low BMI adults, utilizing contemporary commodity prices and historical health outcomes specific to a given population. Malnourished adults represent a particularly vulnerable population in critical need of innovative interventions to improve early ART outcomes, and cost-effectiveness targets for clinical studies are a means to ensure limited health resources can be used to greatest benefit.

## Competing interests

The authors declare that they have no competing interests.

## Authors’ contributions

JK, EM, MG, BC, and JS were responsible for study design and data collection. JS was the program director. MG was the study statistician. JK, EM, and JS constructed the Markov models. JK, EM, MG, BC, and DH drafted the manuscript, which all authors subsequently reviewed, edited and approved.

## Supplementary Material

Additional file 1: Table S1 Sensitivity analysis for the effect of antiretroviral therapy program costs ($500-$1200/patient-year) on nutritional supplement cost-effectiveness, assuming a 20% survival and 20% retention benefit with the intervention. Click here for file

Additional file 2: Figure S1 Maximum permitted quarterly nutritional supplement cost according to antiretroviral therapy program costs. Model assumes 20% mortality and 20% loss to follow-up reduction over 6 months with nutritional supplementation. Click here for file

Additional file 3: Table S2 Sensitivity analysis for the effect of patients classified as lost to follow-up who subsequently return to antiretroviral therapy program on nutritional supplement cost-effectiveness, assuming a 20% survival and 20% retention benefit with the intervention. Click here for file

Additional file 4: Figure S2 Maximum permitted quarterly nutritional supplement cost according to proportion of patients classified as lost to follow-up at 6 months who subsequently return to the antiretroviral therapy program. Model assumes program cost of $556 per patient-year, and 20% mortality and 20% loss to follow-up reduction over 6 months with nutritional supplementation. Click here for file
