# Supplementary material for: Estimating the cost-effectiveness of nutrition supplementation for malnourished, HIV-infected adults starting antiretroviral therapy in a resource-constrained setting
Source: Cost Eff Resour Alloc. 2014 Apr 27;12:10. doi: 10.1186/1478-7547-12-10 (PMC4024113; doi:10.1186/1478-7547-12-10)
Supplement: Additional file 1: Table S1 — Sensitivity analysis for the effect of antiretroviral therapy program costs ($500-$1200/patient-year) on nutritional supplement cost-effectiveness, assuming a 20% survival and 20% retention benefit with the intervention. [file 1478-7547-12-10-S1.doc]

| **Supplementary Table 1:** Sensitivity analysis for the effect of antiretroviral therapy program costs ($500-$1200/patient-year) on nutritional supplement cost-effectiveness, assuming a 20% survival and 20% retention benefit with the intervention. | | |
| --- | --- | --- |
| **Body Mass Index <16.0 kg/m^2^** | | |
| Quarterly ART program costs per patient (USD) | Willingness-to-pay value (USD)* | Maximum permitted quarterly supplement cost (USD) |
| 125 | 767.36 | 9.93 |
| 150 | 920.84 | 11.91 |
| 175 | 1074.31 | 13.90 |
| 200 | 1227.78 | 15.88 |
| 225 | 1381.25 | 17.87 |
| 250 | 1534.73 | 19.86 |
| 275 | 1688.20 | 21.84 |
| 300 | 1841.67 | 23.83 |
|  | | |
| **Body Mass Index 16.00-16.99 kg/m^2^** | | |
| Quarterly ART program costs per patient (USD) | Willingness-to-pay value (USD)* | Maximum permitted quarterly supplement cost (USD) |
| 125 | 762.17 | 5.87 |
| 150 | 914.60 | 7.41 |
| 175 | 1067.03 | 8.64 |
| 200 | 1219.47 | 9.89 |
| 225 | 1371.90 | 11.11 |
| 250 | 1524.33 | 12.35 |
| 275 | 1676.76 | 13.58 |
| 300 | 1829.20 | 14.82 |
|  | | |
| **Body Mass Index 17.00-18.49 kg/m^2^** | | |
| Quarterly ART program costs per patient (USD) | Willingness-to-pay value (USD)* | Maximum permitted quarterly supplement cost (USD) |
| 125 | 760.68 | 4.75 |
| 150 | 912.81 | 5.70 |
| 175 | 1064.95 | 6.65 |
| 200 | 1217.08 | 7.60 |
| 225 | 1369.22 | 8.55 |
| 250 | 1521.35 | 9.50 |
| 275 | 1673.49 | 10.45 |
| 300 | 1825.62 | 11.40 |
| *Represents the cost-effectiveness of ART treatment alone | | |
