# Supplementary figures and images for: Estimating the cost-effectiveness of nutrition supplementation for malnourished, HIV-infected adults starting antiretroviral therapy in a resource-constrained setting
Source: Cost Eff Resour Alloc. 2014 Apr 27;12:10. doi: 10.1186/1478-7547-12-10 (PMC4024113; doi:10.1186/1478-7547-12-10)

## Slide 1
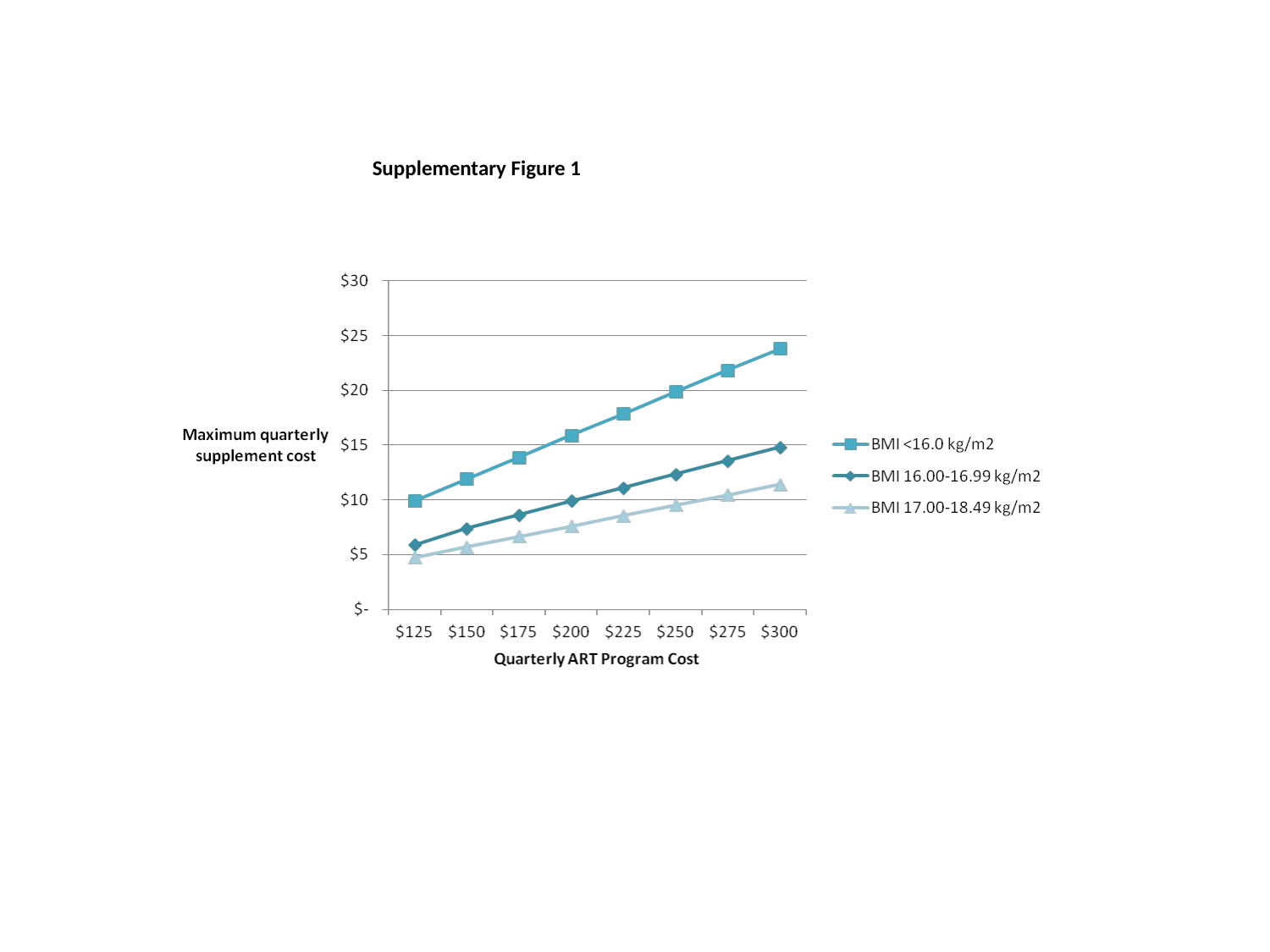

Supplementary Figure 1

Supplement: Additional file 2: Figure S1 — Maximum permitted quarterly nutritional supplement cost according to antiretroviral therapy program costs. Model assumes 20% mortality and 20% loss to follow-up reduction over 6 months with nutritional supplementation. [file 1478-7547-12-10-S2.ppt]

## Slide 1
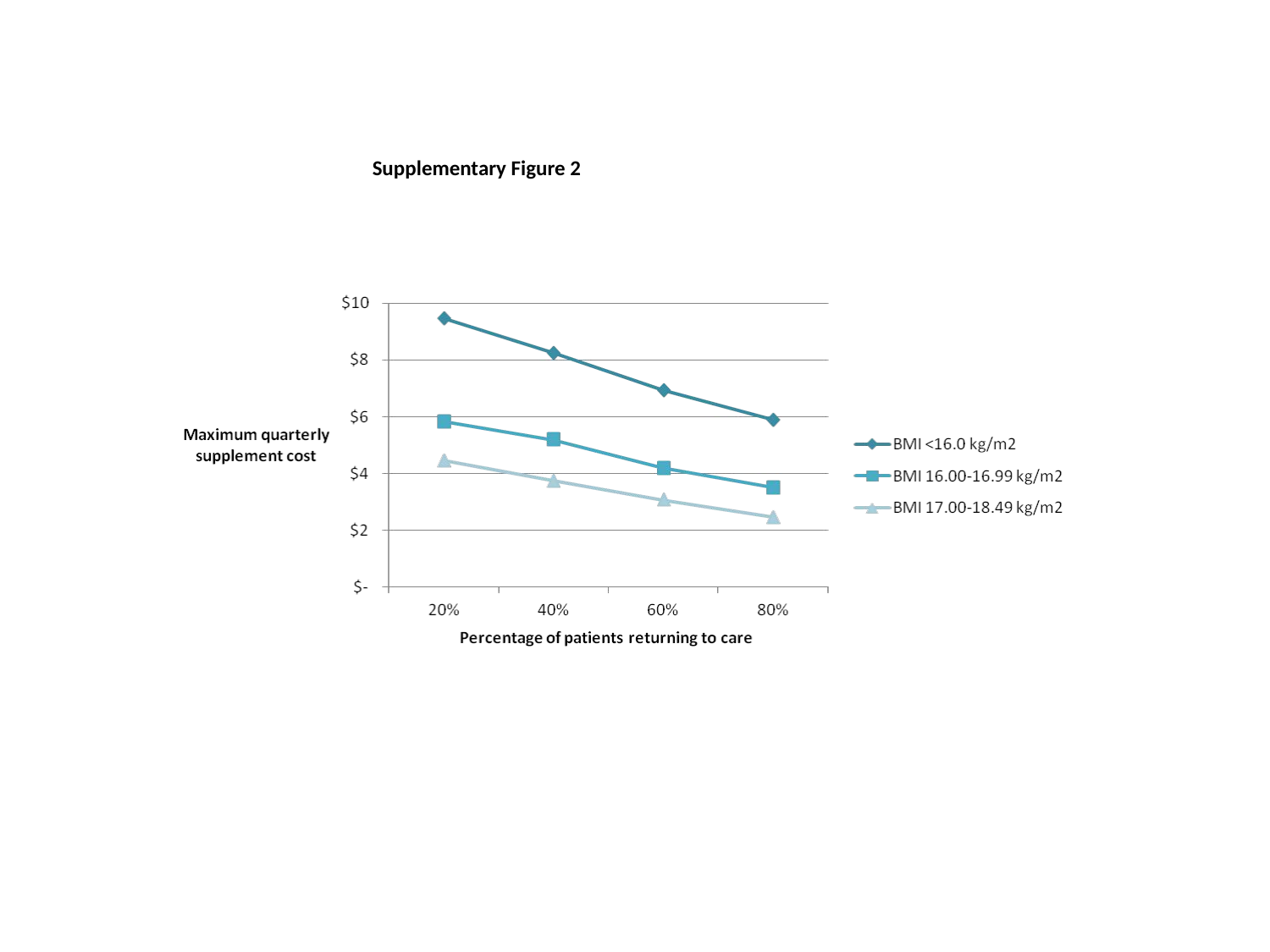

Supplementary Figure 2

Supplement: Additional file 4: Figure S2 — Maximum permitted quarterly nutritional supplement cost according to proportion of patients classified as lost to follow-up at 6 months who subsequently return to the antiretroviral therapy program. Model assumes program cost of $556 per patient-year, and 20% mortality and 20% loss to follow-up reduction over 6 months with nutritional supplementation. [file 1478-7547-12-10-S4.ppt]
