# Supplementary material for: Estimating the cost-effectiveness of nutrition supplementation for malnourished, HIV-infected adults starting antiretroviral therapy in a resource-constrained setting
Source: Cost Eff Resour Alloc. 2014 Apr 27;12:10. doi: 10.1186/1478-7547-12-10 (PMC4024113; doi:10.1186/1478-7547-12-10)
Supplement: Additional file 3: Table S2 — Sensitivity analysis for the effect of patients classified as lost to follow-up who subsequently return to antiretroviral therapy program on nutritional supplement cost-effectiveness, assuming a 20% survival and 20% retention benefit with the intervention. [file 1478-7547-12-10-S3.doc]

| **Supplementary Table 2:** Sensitivity analysis for the effect of patients classified as lost to follow-up who subsequently return to antiretroviral therapy program on nutritional supplement cost-effectiveness, assuming a 20% survival and 20% retention benefit with the intervention. | | |
| --- | --- | --- |
| **Body Mass Index <16.0 kg/m^2^** | | |
| Percentage of patients lost to follow-up who later return to care | Willingness-to-pay value (USD)* | Maximum permitted quarterly supplement cost (USD) |
| 20% | 850.75 | 9.46 |
| 40% | 849.39 | 8.24 |
| 60% | 847.44 | 6.93 |
| 80% | 846.53 | 5.88 |
|  | | |
| **Body Mass Index 16.00-16.99 kg/m^2^** | | |
| Percentage of patients lost to follow-up who later return to care | Willingness-to-pay value (USD)* | Maximum permitted quarterly supplement cost (USD) |
| 20% | 845.53 | 5.82 |
| 40% | 845.65 | 5.19 |
| 60% | 843.37 | 4.18 |
| 80% | 843.08 | 3.49 |
|  | | |
| **Body Mass Index 17.00-18.49 kg/m^2^** | | |
| Percentage of patients lost to follow-up who later return to care | Willingness-to-pay value (USD)* | Maximum permitted quarterly supplement cost (USD) |
| 20% | 844.27 | 4.46 |
| 40% | 843.10 | 3.73 |
| 60% | 842.35 | 3.07 |
| 80% | 841.96 | 2.45 |
| *Represents the cost-effectiveness of ART treatment alone | | |
